# Immunomodulatory Effects of Asiaticoside Against *Shigella flexneri*-Infected Macrophages

**DOI:** 10.21315/tlsr2021.32.2.3

**Published:** 2021-06-29

**Authors:** Shalini Michael, Nor Munirah Zakaria, Muhammad Adamu Abbas, Hasmah Abdullah, Rapeah Suppian

**Affiliations:** 1 School of Health Sciences, Universiti Sains Malaysia, Health Campus, 16150 Kubang Kerian, Kota Bharu, Kelantan, Malaysia; 2 Department of Medical Microbiology and Parasitology, College of Health Sciences, Bayero University Kano, P.M.B. 3011, Kano, Nigeria

**Keywords:** Asiaticoside, Cytokine, Immunomodulatory, Macrophage, Nitric Oxide, Asiatikosid, Sitokin, Imunomodulatori, Makrofaj, Nitrik Oksida

## Abstract

Macrophages provide the first line of defense against *Shigella flexneri* infection in the gastrointestinal tract by inducing a variety of inflammatory and antimicrobial responses. Secondary metabolites of plants are used as drugs against infections that are resistant to common antibiotics. In this study, the innate effects of asiaticoside on the proinflammatory activity of mouse macrophages infected with *S. flexneri* were investigated. The viability of the infected mouse macrophages were examined using viability assay, while the pro-inflammatory cytokines productions were determined using the enzyme-linked immunosorbent assay (ELISA) for determination of IL-1β, IL-12 p40 and TNF-α levels. The production of nitric oxide (NO) and the expression of inducible nitric oxide synthase (iNOS) protein were determined using the Griess assay and western blot, respectively. Statistical analyses were performed using the Statistical Package of Social Sciences (SPSS) software, version 20. The data obtained from independent experiments (*n* = 3) were presented as the mean ± standard error of mean (SEM). The results showed that, asiaticoside stimulated the infected macrophages by stimulating increased production of TNF-α, IL-12 p40 and NO as well as increased expression of iNOS in a dose-dependent manner. In contrast the viability of the cells and the production of IL-1β and were reduced also in a dose-dependent manner when compared to untreated cells. These results indicate that asiaticoside has immunomodulatory effects on the innate immune function of infected macrophages, showing the potential use of this compound to reduce the clinical symptoms of the infections.

## Abstract (fr)

Makrofaj bertindak sebagai sistem pertahanan awal terhadap jangkitan *Shigella flexneri* di dalam saluran gastrousus (saluran pencernaan) dengan mengaktifkan gerak balas inflamatori dan anti-mikrob. Banyak metabolit sekunder daripada tumbuhan digunakan sebagai agen untuk merawat penyakit yang rintang terhadap antibiotik. Dalam kajian ini, kesan gerak balas semulajadi asiatikosid terhadap aktiviti pro-inflamasi makrofaj mencit yang telah dijangkiti *S. flexneri* telah dikaji. Kedayahidupan makrofaj mencit dianalisis menggunakan asai kedayahidupan, manakala penghasilan sitokin pro-inflamasi IL-1β, IL-12 p40 dan TNF-α dianalisis menggunakan esei imunoserapan terangkai enzim (ELISA). Penghasilan nitrik oksida (NO) dan pengekspresian protein sintase niktrik oksida teraruh (iNOS) dianalisis menggunakan asai Griess dan analisis pemblotan Western. Analisis statistik dijalankan menggunakan perisian ‘Statistical Package of Social Sciences (SPSS)’ versi 20. Data yang diperolehi daripada eksperimen tak bersandar (*n* = 3) dibentangkan sebagai min ± min ralat piawai (SEM). Hasil kajian menunjukkan, asiatikosid merangsang pengaktifan makrofaj mencit yang telah dijangkiti melalui peningkatan penghasilan TNF-α, IL12 p40 dan NO serta meningkatkan penghasilan iNOS bergantung kepada dos asiatikosid yang diberikan. Namun begitu, kedayahidupan sel dan penghasilan IL-1β menurun seiring dengan dos yang diberikan dalam sel yang dirawat berbanding dengan sel yang tidak dirawat. Hasil kajian ini menunjukkan bahawa asiatikosid mempunyai kesan imunomodulatori terhadap fungsi imun semulajadi makrofaj yang dijangkiti, yang memberi gambaran potensinya untuk digunakan bagi mengurangkan simptom klinikal jangkitan *S. flexneri*.

**Highlights**

- The viability of the infected mouse macrophages were examined using viability assay, the pro-inflammatory cytokines productions were determined using the enzyme-linked immunosorbent assay (ELISA) for determination of IL-1β, IL-12 p40 and TNF-α levels, the production of nitric oxide (NO) and the expression of inducible nitric oxide synthase (iNOS) protein were determined using the Griess assay and western blot, respectively.
- Asiaticoside stimulated the infected macrophages by stimulating increased production of TNF-α, IL-12 p40 and NO as well as increased expression of iNOS in a dose-dependent manner.
- Asiaticoside has immunomodulatory effects on the innate immune function of infected macrophages, showing the potential use of this compound to reduce the clinical symptoms of the infections.

## INTRODUCTION

Gastroenteritis, one of the most important causes of paediatric morbidity and mortality worldwide (Farthing *et al*. 2012) is a result of the complex inflammatory processes induced by antigens of invading organisms (Stromberg *et al*. 2018) activated by nuclear transcription factor NF-kB, which is a promoter of pro-inflammatory cytokine production and recruitment of neutrophils, Polymorphonuclear neutrophil (PMN) and macrophages at the sites of infection (Liu *et al*. 2017). Macrophages phagocytose invading organisms and have the ability to secrete pro-inflammatory cytokines such as tumour necrosis factor-α (TNF-α), interleukin-1 (IL-1), interleukin-12 (IL-12) and toxic mediators such as nitric oxide (NO) against invading pathogens (Hirayama *et al*. 2018). They are also responsible for maintaining the integrity of the epithelial barrier, regulatory T cells in the mucosal surface (Bording-Jorgensen *et al*. 2018) and tissue homeostasis (Zhao *et al*. 2018).

*Shigellosis*, a diarrheal disease caused by one of the four serogroups of *Shigella* species, namely: *S. flexneri, S. dysenteriae, S. boydii* and *S. sonnei*. *Shigellosis* leads to about 125 million episodes of diarrhoea annually (Bardhan *et al*. 2010). Treatment for *Shigellosis* is hampered by its resistance to fluoroquinolones (Puzari *et al*. 2018), azythromycin (Zhang *et al*. 2017) and cephalosporins (Mahmoudi *et al*. 2017) and multidrug (Dhital *et al*. 2017) leading to the search for plant products (Mirza *et al*. 2018). Asiaticoside, a compound from *Centella asiatica* was reported to have antibacterial (Haralampidis *et al*. 2002) anti-inflammatory (Guo *et al*. 2004), wound healing (Omar *et al*. 2017) and immune stimulating properties (Ansari & Inamdar 2010). However, knowledge of its effect on *S. flexneri* infection is still lacking. Using the J774A.1 mouse macrophage cells, the present study was carried out to determine the effects of asiaticoside on the cell viability, expression of iNOS, the production of NO, and pro-inflammatory cytokines such as TNF-α, IL-1β and IL-12 when infected with *S. flexneri*.

## MATERIALS AND METHODS

### Bacterial and Cell Culture

The Culture Laboratory of the School of Health Sciences, Universiti Sains Malaysia provided the bacterial strains of *S. flexneri*. The strains were then streaked on blood agar under a sterile condition and incubated overnight at 37°C. A single colony of *S. flexneri* was then picked using a sterile loop and transferred into Mueller Hinton (MH) broth. The broth was incubated at 37°C in a shaking incubator, then the bacterial cells were harvested, optical densities (OD) measured and multiplicities of infection (MOI) determined for co-culture with macrophages. J774A.1 mouse macrophage cell lines purchased from the American Type Culture Collection (ATCC) were cultured in Dulbecco’s Modified Eagle Medium (DMEM) (Sigma, USA) supplemented with 100 μg/mL Penicillin and 10% fetal bovine serum (Sigma, USA). The cells were incubated at 37°C in a humidified atmosphere of 5% CO_2_ overnight before being treated with different concentrations of asiaticoside.

### Preparation of Asiaticoside

Asiaticoside (Sigma, USA) was dissolved in 1:10 Dimethyl sulfoxide (DMSO) and diluted to 1 mg/mL stock solution. In order to evaluate the effect of the compound on the macrophages, the stock was then further diluted to different concentrations within the range of 0, 10, 50 and 100 μg/mL.

### Infection of Murine Macrophage Cell Line J774A.1 with *S. flexneri*

Macrophages (5 × 10^5^ cells/mL) were treated with varying concentrations of asiaticoside (0,10, 50 and 100 μg/mL). The cells were then incubated in a humidified 5% CO_2_ incubator for an hour before being infected with 1 × 10^8^ CFU *S. flexneri* and further incubated at 37°C for 24 h. Uninfected macrophage cells served as a negative control while those treated with LPS (100 ng/mL) was used as a positive control (Cocco & Ucker 2001). The cell culture solutions were then centrifuged at 1 500 x g for 10 min at room temperature and the supernatants and pellets were used for cytokine and protein determination.

### Macrophage Viability

Cell viability was determined by MTT 3-(4,5-dimethylthiazol-2-yl)-2,5-diphenyltetrazolium bromide) assay as following the manufacturer’s protocol. Briefly, cells (1 × 10^5^) were cultured and treated in a 96 well plate for 24 h. The cells were then washed with PBS, resuspended in fresh DMEM then 10 μL of 12 mM MTT stock solution was added and incubated at 37°C for 4 h. Next, 100 μL of SDS-HCl solution was added into each well and mixed thoroughly and the plate was incubated for 4 h at 37°C. The absorbance was measured using a microplate reader at 570 nm.

### Measurement of Pro-inflammatory Cytokine

The supernatants of the infected cells and the controls collected after 24 h incubation were used to determine the production of TNF-α and IL-12 p40 using their respective ELISA kits (Biolegend, UK) while the production of IL-1β was determined using an IL-1β ELISA kit (eBioscience, UK). Briefly, the antibody of IL-1β, TNF-α or IL-12 p40 were used in coating a 96 well ELISA plate and incubated overnight at 4°C. The plate was washed 5 times with PBS-T20 prior to blocking with blocking buffer for 1 h at room temperature. This was then followed by 5 washes again. Next, 100 μL of the culture supernatant and standard were added to corresponding wells, sealed and incubated for another 2 h at room temperature then washed again. This was then followed by the addition of 100 μL of respective detection antibodies, then incubation at room temperature for 1 h. The plate was then washed, and then 100 μL Avidin HRP added with 30 min incubation followed by 5 washes. A substrate solution was added and the solution incubated at room temperature for 15 min in the dark. Stop solution was then added and the plate was read by a microplate reader at 450 nm to determine cytokine concentration and generate a standard curve.

### Determination of Nitric Oxide (NO) Production

The production of NO was determined in the culture supernatant of the cells using the Griess reagent kit (Promega, USA) based on the manufacturer’s protocol. Briefly, 50 μL of the standards and samples were added to the 96 well plates before dispensing 50 μL sulphanilamide solutions. This was incubated for 5 to 10 min at room temperature, in dark after which 50 μL NED solutions was added and incubated in the dark at room temperature for 5 to 10 min. The plates were read at 540 nm using a microplate reader and a standard curve was generated.

### Determination of iNOS Expression

The pellets of the infected cells and the controls collected after 24 h incubation were washed and lysed with ice-cold RIPA buffer. The lysates were centrifuged at 13 000 x g for 15 min followed by denaturing of the supernatants using Laemmli buffer, and fractionation on 12% (w/v) polyacrylamide gels using electrophoresis. This is followed by transfer to polyvinylidene difluoride (PVDF) membrane. The membrane was then blocked with 1% skimmed milk solution for 1 h before incubation with rabbit anti-mouse iNOS antibody for 2 h at 37°C followed by incubation with goat anti-mouse antibody conjugated to HRP for 1 h at 37°C. Chemiluminescence was generated by an ECL Western blot detection reagent. An antibody specific to β-actin was used as a control. Integrated density of the iNOS band was quantified using Image J software and normalised with that of β-actin.

### Statistical Analysis

Statistical analyses were carried out using the Statistical Package of Social Sciences (SPSS) software, version 20. The data obtained from independent experiments (*n* = 3) were presented as the means ± standard error of the mean (SEM). A paired two-tailed student’s *t*-test was used to determine the statistical significance between the treated and untreated cells. We considered *P*-value of <0.05 as statistically significant.

## RESULTS

### Effects of Asiaticoside on Cell Viability

**Figure 1:**
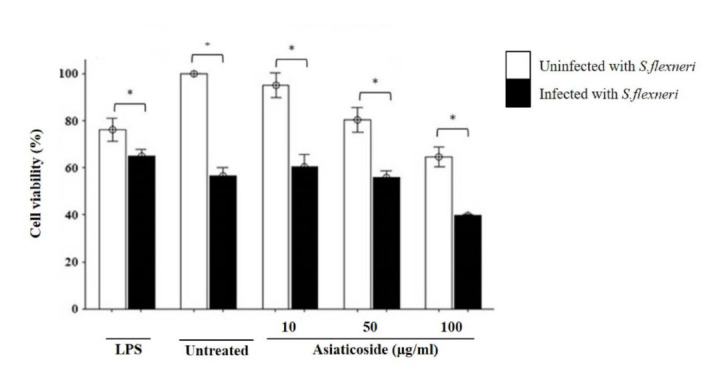
Effects of asiaticoside on the cell viability of J774A.1 mouse macrophages cell line infected with *S. flexneri* in the presence or absence of asiaticoside. The viability of the cells was evaluated using Vybrant MTT Cell Proliferation Assay kit (Thermo Fisher Scientific, USA). Values are expressed as the mean ± SEM for three independent experiments. *p < 0.05 is considered significant.

The effects of different concentrations of asiaticoside on the viability of macrophage infected with *S. flexneri* were determined using cell viability assay. Our data showed that macrophage viability reduced significantly when treated with the concentrations of asiaticoside in the presence of *S. flexneri* infection (Fig. 1). When the macrophages groups were compared, the highest reductions were found in the *S. flexneri* infected macrophage group. *S. flexneri* led to decrease viability in macrophages treated with LPS compared to uninfected macrophages with the same treatment. Cell viability was highest in uninfected unstimulated cells and is lowest with the highest dose of asiaticoside.

### Effect of Asiaticoside on Cytokine Production

**Figure 2:**
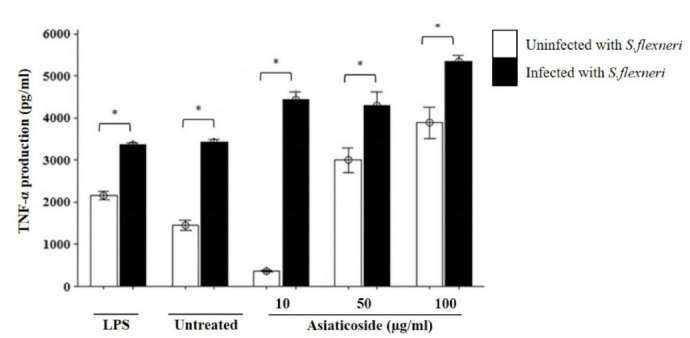
Effects of asiaticoside on TNF-α production in J774A.1 mouse macrophages cell line infected with *S. flexneri* in the presence or absence of asiaticoside. Values are expressed as the mean ± SEM for three independent experiments. * p < 0.05 is considered significant.

The immunomodulatory effects of asiaticoside of some pro-inflammatory cytokines were analysed. When TNF-α production was analysed, there was a significant increase in a dose dependent manner especially in the *S. flexneri*-infected cells treated with asiaticoside (Fig. 2). In contrast to cell viability, the production of TNF-α was reduced in the uninfected cell of 10 μg/ml asiaticoside which gradually increased with increased concentration of asiaticoside, while a uniform dose-dependent increase was observed in the *S. flexneri* infected cells. In both infected and uninfected cells, the highest production of TNF-α was in the cells treated with the 100 μg/mL asiaticoside. On treatment with LPS, TNF-α production was higher in the *S. flexneri*-infected macrophages compared to un-infected macrophages similar to the unstimulated cells.

**Figure 3:**
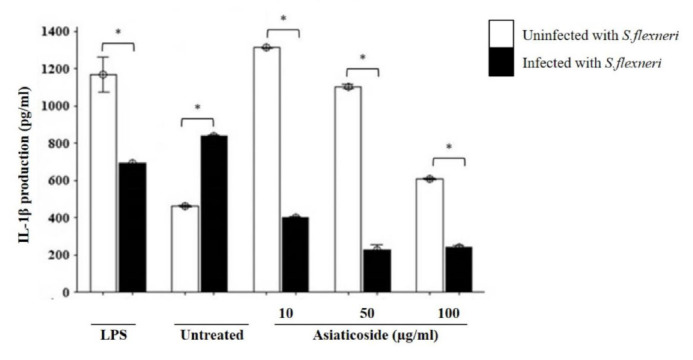
Effects of asiaticoside on IL-1β production in J774A.1 mouse macrophages cell line infected with *S. flexneri* in the presence or absence of asiaticoside. Values are expressed as the mean ± SEM for three independent experiments. * p < 0.05 is considered significant.

The production of IL-1β was also measured. The highest level was detected in uninfected macrophages treated with 10μg/ml asiaticoside (Fig. 3). Treatment with asiaticoside led to a significant dose-dependent decrease in the productions of IL-1β in uninfected cells. The asiaticoside treatment also led to a significant inhibition the production of IL-1β in the macrophage infected with *S. flexneri* compared to the uninfected. When stimulated with LPS, IL-1β production was also significantly lower in the infected macrophages compared to the uninfected ones, while the production significantly higher in the unstimulated macrophages infected with *S. flexneri* compared to the uninfected.

**Figure 4:**
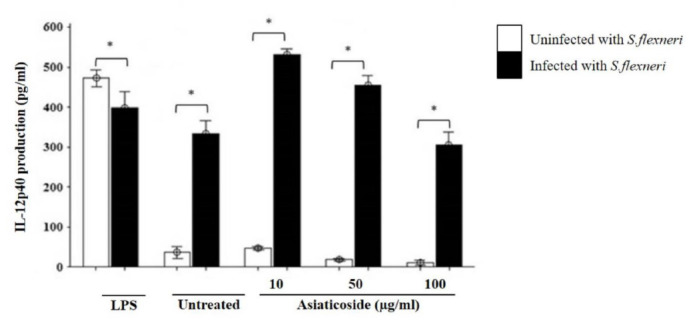
Effects of asiaticoside on IL12 p40 production in J774A.1 mouse macrophages cell line infected with *S. flexneri* in the presence or absence of asiaticoside. Values are expressed as the mean ± SEM for three independent experiments. * p < 0.05 is considered significant.

Infecting macrophages with *S. flexneri* also led to significant production of IL-12 p40. The production of IL12 p40 in both infected and uninfected macrophages was increased on treatment with asiaticoside (Fig. 4). This increase was more enhanced with the minimum concentration in asiaticoside in both groups, being highest on treatment with 10 μg/mL asiaticoside and most reduced with 100 μg/mL asiaticoside. Both unstimulated macrophages and those stimulated with LPS showed robust production of IL12 p40 which was significantly higher in the infected cells.

### Effects of Asiaticoside on the NO

**Figure 5:**
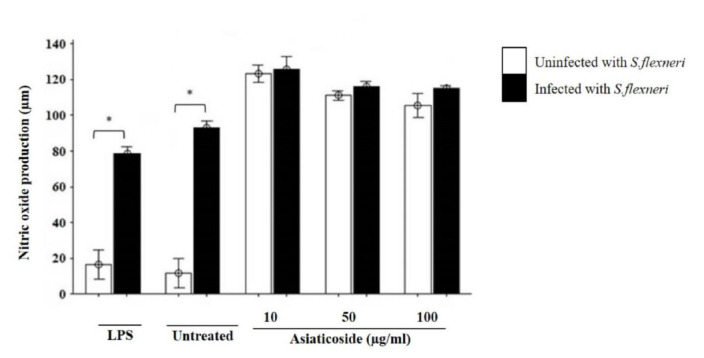
Effects of asiaticoside on NO production in J774A.1 mouse macrophages cell line infected with (*S. flexneri* in the presence or absence of asiaticoside. Values are expressed as the mean ± SEM for three independent experiments. * p < 0.05 is considered significant.

The NO production was also analysed and was found to be higher in the 10 μg/mL asiaticoside treated cells in the presence and absence of *S. flexneri* (Fig. 5). The production of NO was significantly increased in the untreated cells and those stimulated by LPS, in response to *S. flexneri*. NO production was highest with the minimum dose (10 μg/mL) of asiaticoside which was uniformly reduced with an increased dose of asiaticoside in both *S. flexneri*-infected and uninfected cells. However, these increases were not significantly different in both the infected and uninfected cells. In the LPS treated and the unstimulated cells, there were significant increases in NO production in the *S. flexneri*-infected cells.

### Effects of Asiaticoside on the Productions of Inducible Nitric Oxide Synthase (iNOS)

**Figure 6:**
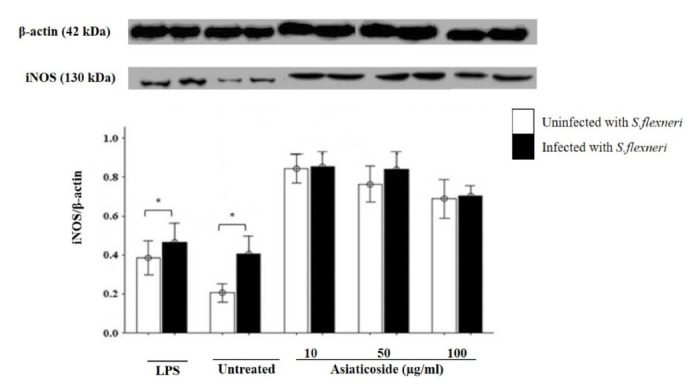
Effects of asiaticoside on iNOS production in J774A.1 mouse macrophages cell line infected with *S. flexneri* in the presence or absence of asiaticoside. Values are expressed as the mean ± SEM for three independent experiments. * *p* < 0.05 is considered significant compared with the uninfected group.

Western blot analysis was carried out to determine the expression of iNOS in the presence or absence of *S. flexneri* infections. The results showed that iNOS protein was expressed in all cells, treated or untreated. The expression increased when macrophages, infected and uninfected, were treated with 10 μg/mL asiaticoside. However, this expression was reduced with increased concentration of asiaticoside. Similar to NO production, the iNOS production was not significantly different between asiaticoside-treated cells, but significantly different in the control groups the expression was increased in the presence of *S. flexneri* (Fig. 6).

## DISCUSSION

Macrophages are specialised scavenger cells (Bellomo *et al*. 2018) that regulate innate (Perez *et al*. 2017) and adaptive (Navegantes *et al*. 2017) immune responses. They control invading pathogens via phagocytosis (Bardhan *et al*. 2010) and activation of other immune cells through secretion of pro-inflammatory cytokines (Chiu & Bharat 2016)*. Shigella*, an enteroinvasive bacterium that resides in macrophages survive in the host and produces effectors that down-regulate the innate immune responses (Mattock & Blocker 2017). Studies have shown that gastrointestinal infections caused by *Shigella* spp. induce the production of proinflammatory cytokines (Gopal *et al*. 2017) such as TNF-α, IL-1β and IL12 p40 as part of the host immune response. Plants such as *C. asiatica* and its active compound, asiaticoside have been used to treat gastrointestinal disorders. This study investigated the immunomodulatory effects of asiaticoside against *S. flexneri*-infected macrophages. The results obtained showed that asiaticoside was able to stimulate an inflammatory response in macrophages in response to *S. flexneri* infection. There was increased production of TNF-α, IL12 p40, NO and iNOS in the supernatant of the cells infected with *S. flexneri* and a general reduction in cell viability in macrophages.

Our results showed that cell viability was higher in the uninfected macrophages, but reduced in the *S. flexneri*-infected cells in a dose-dependent manner. The mechanism by which asiaticoside inhibited the growth of the bacteria and causes cell death is not yet known, but previous studies have reported a correlation between the cytotoxic activity of plants or plant products and cell viability (Robinson *et al*. 2017). This could be as a result of the production of reactive oxygen and nitrogen intermediates, and possibly acidification of phagosomes by the infected macrophages (Kyriazis *et al*. 2016). It could also involve macrophage apoptosis (Frankenberg *et al*. 2008).

This study also looked into the production of TNF-α, a pleiotropic proinflammatory cytokine expressed in many human diseases including gastrointestinal infection. Robust production of TNF-α was observed in all *S. flexneri-*infected macrophages with a significantly enhanced production in the presence of asiaticoside, also in a dose-dependent manner. Similar to these findings, studies have found enhanced TNF-α levels in *Shigella* infections (McArthur *et al*. 2017) and *Shigella* outer membrane vesicle-based vaccines (Mitra *et al*. 2015). Asiaticoside has been shown to have immunomodulatory properties against *Leishmaniasis* where it induced TNF-α production in peritoneal macrophages (Bhaumik *et al*. 2012). TNF-α has an effect on the activity of basolateral membrane, causing an increment of chloride (Cl^−^) secretion and decreases the absorption of natrium (Na^+^) in epithelial cells leading to accumulation of fluid in the small intestine that induced diarrhoea (Mattock & Blocker 2017). This high-volume fluid accumulation in the intestine is essential in bacterial clearance and reduction of colonisation of the infected pathogen (Boudry *et al*. 2004). Therefore, higher TNF-α production by the macrophages in response to asiaticoside in this study indicated the capability of the product to modulate the cells to eliminate the pathogens. The presence of rhamnose sugar in asiaticoside makes it a potent macrophage activator leading to TNF-α induction (Bhaumik *et al*. 2012).

We also investigated the production of IL-1β and found it to be increased in uninfected macrophages cells. Interestingly, rather than IL-1β production to increase in *S. flexneri* infection, it was reduced. IL-1β was further reduced in a dose-dependent manner in the presence of asiaticoside in both infected and uninfected macrophages, being significantly so in the infected cells. This data was in agreement with Mitra, who found no effects on IL-1β production in mice immunised with *Shigella* vesicle-based vaccine (Mitra *et al*. 2015). It has also been reported that IL-1β expressions and release into the basal layer of the gastrointestinal tract is induced by macrophage apoptosis and inflammation against *S. flexneri* (Yang *et al*. 2015), while other studies reported an increase in macrophage apoptosis with reduced production of IL-1β modulated by asiaticoside (Al-Saeed 2014), a possible explanation to the observed decreased cell viability in the infected cells and decreased IL-1β production in a dose-dependent manner in this study.

The production of IL12 p40 was also analysed due to its importance in bacterial infection since *Shigella* infections lead to an increase in the IL12 p40 production of macrophages. Expectedly, our results showed significant production of IL12 p40 in *S. flexneri-*infected macrophages. This was similar to a rabbit illeal loop *S. flexneri* infection model which showed significant induction of IL12 p40 production (Schnupf & Sansonetti 2012). Our result further showed that asiaticoside in the presence of *S. flexneri* led to maximum production of IL12 p40 at 10 μg/mL, while increasing its concentration resulted in reduced IL12 p40 production, indicating its immunomodulatory effects at a low dose.

The production of NO is dependent on the expression of the enzyme iNOS. This production is mediated via a series of signaling events like activation of NF-κβ and mitogen-activated protein (MAP) kinases (Hayden *et al*. 2006). Our results showed increased production of both NO and iNOS in *S. flexneri* infection, with maximum, though not significant production at 10 μg/mL asiaticoside in both infected and uninfected macrophages. Some studies have also found increased production of NO by asiaticoside (Wang *et al*. 2017) while others found a reduction in both NO and iNOS when SH-SY5Y cell lines exposed to H_2_O_2_-induced toxicity where treated with asiaticoside (Ling *et al*. 2017). Asiaticoside in *Leishmaniasis* induced the production of NO in peritoneal macrophages depending on concentration (Bhaumik *et al*. 2012). Oleuropein exhibited similar effects on splenocytes and hepatocytes cell cultures (Kyriazis *et al*. 2016). It has been postulated that treating macrophages with a higher concentration of asiaticoside may lead to a reduction in the degradation of IκB-α and mobilisation of p65 into the nucleus of the macrophages, thus leading to the reduction in the activation of NF-κβ. However, this reduction was probably only sufficient for the induction of TNF-α production but not enough for the induction of complete iNOS expression (Eswarappa *et al*. 2008), hence the observed pattern in this study.

Essentially, the results from this study highlighted the immunomodulatory effects of asiaticoside against *S. flexneri*-infected macrophages. We assume that the presence of asiaticoside might be responsible for this phenomenon as it possesses wound healing and inflammatory properties (Mahomoodally 2013). These effects were significantly highest at the dose of 10 μg/mL asiaticoside and tend to wane down with increased dosage. This and the fact that asiaticoside decreased IL-1β point to the possibility of induction of anti-inflammatory cytokines by the extract. Further insight into these effects could be made through the studies of the induction of anti-inflammatory cytokines on treatment with asiaticoside as the need for extensive researches on ethnomedicines are always desirable for evidence-based usage.

## CONCLUSION

We demonstrated that asiaticoside has potential immunomodulatory effects on macrophages infected with *S. flexneri* in a dose-dependent manner. These effects are important for the elimination of bacteria during intestinal infections. Thus, taking this compound as a dietary supplement at a suitable dose could enhance the effectiveness of macrophages to kill the pathogens during phagocytosis, an important stage in infection prevention and control. Asiaticoside can also be used to reduce the possible harmful effects of the infections due to the excessive amount of toxic mediators such as NO due to its probable release of regulatory cytokines.
